# Phase II trial of topically applied miltefosine solution in patients with skin-metastasized breast cancer

**DOI:** 10.1038/sj.bjc.6690184

**Published:** 1999-03

**Authors:** J M M Terwogt, I A M Mandjes, H Sindermann, J H Beijnen, W W ten Bokkel Huinink

**Affiliations:** Department of Medical Oncology, The Netherlands Cancer Institute/Antoni van Leeuwenhoek Huis, Plesmanlaan 121, 1066 CX, Amsterdam, The Netherlands; Department of Pharmacy and Pharmacology, The Netherlands Cancer Institute/Slotervaart Hospital, Louwesweg 6, 1066 EC, Amsterdam, The Netherlands; ASTA Medica AG, Weismüllerstraβe 45, P.O. Box 10 01 05, 6000, Frankfurt am Main 1, Germany

**Keywords:** miltefosine, topical administration, skin metastases, breast cancer

## Abstract

Skin deposits from breast cancer can present serious therapeutic problems, especially when resistant to conventional therapy. Topical application of a cytotoxic drug may represent an attractive new treatment modality devoid of major systemic toxicity. Miltefosine was selected because of its efficacy in breast cancer models. A mixture of alkylated glycerols of various chain lengths and water was used as the pharmaceutical vehicle to dissolve and to further facilitate tissue penetration of miltefosine. In our Institute a phase II study was performed to determine the efficacy and tolerability of topically applied miltefosine in patients with cutaneous metastases from breast cancer. Thirty-three patients in total entered the trial. A 6% miltefosine solution was applied once daily in the first week and twice daily in the following weeks. The planned minimum treatment duration was 8 weeks. We found an overall response rate of 43% for 30 evaluable patients, composed of 23% complete response and 20% partial response. The median response duration was 18 weeks, range 8–68. Toxicity consisted mainly of localized skin reactions, which could be controlled by a paraffin-based skin cream and, where appropriate, by dose modification. No systemic toxicities were observed. We conclude that topical miltefosine is an effective treatment modality in patients with skin metastases from breast cancer. © 1999 Cancer Research Campaign


*British Journal of Cancer* (1999) **79**(7/8), 1158–1161
© 1999 Cancer Research Campaign
Article no. bjoc.1998.0184

### Phase II trial of topically applied miltefosine solution in patients with skin-metastasized breast cancer

**JMM Terwogt[1,2], IAM Mandjes[1], H Sindermann[3], JH Beijnen[1,2] and WW ten Bokkel Huinink[1]**

[1]Department of Medical Oncology, The Netherlands Cancer Institute/Antoni van Leeuwenhoek Huis, Plesmanlaan 121, 1066 CX, Amsterdam, The Netherlands; [2]Department of Pharmacy and Pharmacology, The Netherlands Cancer Institute/Slotervaart Hospital, Louwesweg 6, 1066 EC, Amsterdam, The Netherlands; [3]ASTA Medica AG, Weismüllerstraβe 45, P.O. Box 10 01 05, 6000 Frankfurt am Main 1, Germany

**Summary** Skin deposits from breast cancer can present serious therapeutic problems, especially when resistant to conventional therapy. Topical application of a cytotoxic drug may represent an attractive new treatment modality devoid of major systemic toxicity. Miltefosine was selected because of its efficacy in breast cancer models. A mixture of alkylated glycerols of various chain lengths and water was used as the pharmaceutical vehicle to dissolve and to further facilitate tissue penetration of miltefosine. In our Institute a phase II study was performed to determine the efficacy and tolerability of topically applied miltefosine in patients with cutaneous metastases from breast cancer. Thirty-three patients in total entered the trial. A 6% miltefosine solution was applied once daily in the first week and twice daily in the following weeks. The planned minimum treatment duration was 8 weeks. We found an overall response rate of 43% for 30 evaluable patients, composed of 23% complete response and 20% partial response. The median response duration was 18 weeks, range 8–68. Toxicity consisted mainly of localized skin reactions, which could be controlled by a paraffin-based skin cream and, where appropriate, by dose modification. No systemic toxicities were observed. We conclude that topical miltefosine is an effective treatment modality in patients with skin metastases from breast cancer.

**Keywords:** miltefosine; topical administration; skin metastases; breast cancer

Skin metastases often represent a major therapeutic problem in oncology, especially in patients with metastases no longer amenable to standard therapeutic measures such as surgery, radiation or systemic chemotherapy (Unger et al, 1990, 1992). On the other hand, treatment is complicated in situations when skin metastases progress despite response to systemic treatment at other sites (mixed response). The visibility of the tumours is often an additional aggravating factor in the patients' psychological situation. Topical administration of a cytotoxic drug could offer possibilities for local treatment with the additional advantage of avoiding the risk of major systemic toxicity.

Miltefosine (hexadecylphosphocholine, He-PC) is an alkylphosphocholine with proven cytotoxicity in diverse series of rodent and human cell lines (Eibl et al, 1986; Unger et al, 1989) (Figure 1). In vivo, miltefosine has shown anti-tumour activity against chemically induced rat mammary tumours as well as human breast carcinomas (Hilgard et al, 1988; Fichtner et al, 1994). Unlike most other anti-tumour substance, alkylphosphocholines do not attack the cell nucleus but are active at the level of the cell membrane (Eibl and Unger, 1990; Unger and Eibl, 1991). Miltefosine is thought to exert its cytotoxic action predominantly by interacting with the cell membrane components that are linked to phospholipid turnover and membrane signal transduction (Hilgard et al, 1993; Berkovic et al, 1995; Goppelt-Struebe and Winter, 1995). As a result, miltefosine inhibits protein kinase C, which induces cell differentiation (Hilgard et al, 1989). Verweij and others performed a series of phase I and phase II studies with orally administered miltefosine in soft tissue sarcoma, colorectal cancer and squamous cell head and neck cancer patients but, unfortunately, no significant activity was found (Verweij et al, 1992, 1993*a*, 1993*b*; Planting et al, 1993). Nausea and vomiting appeared to be dose-limiting and some patients developed a reversible renal dysfunction.

For topical application, however, miltefosine is a suitable candidate because it can penetrate the skin, although not to an extent causing systemic side-effects. It is pharmaceutically formulated in a mixture of water and 1-O-alkylglycerols, amphiphilic molecules that can change the permeability of membranes as shown by the penetration of cytostatics across the blood–brain barrier (Unger et al, 1984; Eibl and Unger, 1990). Moreover, alkylglycerols with chain lengths >C10 have been shown to possess anti-tumour activity (Ando et al, 1972). Miltefosine in this formulation appeared to be effective topically in the treatment of some cutaneous lymphomas and in progressive skin metastases from breast cancer (Unger et al, 1988; Dummer et al, 1993; Clive and Leonard, 1997). The recommended dose for topical application resulting from a phase I clinical trial with dose-escalation in breast cancer patients was 60 mg ml$^{-1}$ (a miltefosine 6% solution), the quantity of solution

*Received 30 December 1997*
*Revised 18 May 1998*
*Accepted 4 June 1998*

*Correspondence to:* JM Meerum Terwogt, Department of Pharmacy and Pharmacology, Slotervaart Hospital, Louwesweg 6, 1066 EC, Amsterdam, The Netherlands

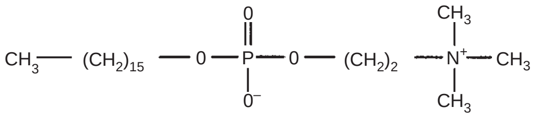

**Figure 1** Structural formula of miltefosine being applied according to the size of the affected area. No systemic side-effects were observed and the dose-limiting toxicity at 80 mg $ml^{-1}$ was erythema of the non-pretreated skin, which was reversible at 60 mg $ml^{-1}$ (Unger et al, 1992). In this paper we report the results of a phase II clinical trial performed in our Institute with topically applied miltefosine in patients with skin-metastasized breast cancer.

### PATIENTS AND METHODS

### Patient population

Patients were eligible if they had a histologically verified breast carcinoma with progressive skin metastases amenable to topical treatment without concomitant systemic anti-tumour therapy. There were no restrictions on volume, depth or ulceration. Previous chemotherapy or radiotherapy was accepted as long as the last administration was at least 4 weeks before the study entry, or 6 weeks in case of pretreatment with nitrosourea or mitomycin C. All patients had a performance status ≤ 2 (WHO) and an estimated life expectancy of ≥ 3 months. Further inclusion criteria were a baseline number of thrombocytes $\geq 100 \times 10^9 l^{-1}$, leukocytes $\geq 2.5 \times 10^9 l^{-1}$ and transaminases ≤ 4 times the upper limit of reference range. The study protocol was reviewed and approved by the Ethics Committee of the Institute and all patients gave written informed consent.

### Treatment plan and study design

A 6% solution of miltefosine in a defined mixture of 3-alkoxy-propyleneglycols (glycerolethers) (Miltex®, ASTA Medica AG, Frankfurt, Germany) was applied once daily for the first week and, if tolerated, twice daily thereafter. The solution was applied to, and gently rubbed into, the lesion and involved skin with the inclusion of a 3 cm margin of surrounding normal skin. The total dose applied therefore varied according to the size of area treated. A mixture of 10% vaseline was applied in addition to the miltefosine lotion to prevent or improve desquamation. The anticipated minimum treatment duration was 8 weeks. In cases of a complete remission, therapy was continued for another 4 weeks and then discontinued, while in cases of no change or partial remission, therapy continued until relapse. After a pretreatment assessment to verify eligibility for the trial patients were reassessed by clinical history, physical examination and laboratory investigations after 1 week, 2 weeks, and every 4 weeks thereafter while on treatment, and at the end of treatment.

### Response criteria

Response was evaluated by measurement of indicator lesions together with a photograph every 4 weeks and at the end of treatment. Patients who had a complete disappearance of all treated lesions for at least 4 weeks were considered to have experienced a complete response. A partial response was defined as an estimated decrease in tumour size of 50% or more for at least 4 weeks. Stable disease included an estimated decrease of lesions of less than 50% and an estimated increase of lesions of less than 25%. Progressive disease was defined as an increase in tumour size of 25% or more. New lesions outside the treated area were not considered as evidence of (local) progression and subsequent inclusion of new (non-indicator) lesions after a response in the primary treated area was allowed.

### Other criteria

Toxicity was classified and graded according to WHO criteria. Response duration was calculated between start of treatment and first observation of progression or treatment failure (time to progression).

### RESULTS

### Patients

Thirty-four female patients with a primary diagnosis of breast cancer were entered into the trial. One patient did not return to hospital after the pretreatment assessment and has therefore been excluded from further analyses. Of the remaining 33 patients, the median age was 57 years with a range of 30–90 years. All had previously received extensive tumour-specific treatment. Table 1 summarizes their main characteristics. Skin lesions were classified as either 'indicator lesions' (lesions located in the started area of miltefosine treatment) or 'non-indicator lesions' (later extensions of the initial treatment area). Twenty-two of the 33 patients (67%) had 1–3 indicator lesions and 11 patients (33%) had more than 3 lesions. The 33 patients were treated with miltefosine for 1–68 weeks and the median treatment duration was 10 weeks. Fourteen patients (42%) were treated for more than 12 weeks. Local progression in the treated area was the most frequent reason to stop further miltefosine treatment (19 patients). Other reasons were systemic progression (nine), local progression outside treated area (four), loss to follow-up (two), adverse skin reactions (two), refusal (one) and poor drug tolerance (one).

### Toxicity

All 33 patients were evaluated for toxicity. Adverse drug reactions (ADRs) were mainly related to skin reactions. Overall, 22 patients (67%) reported adverse skin reactions, predominantly consisting of a dry, erythematous, itching or painful skin, sometimes accompanied by desquamation. In most patients, skin reactions occurred after 3 weeks of treatment. The maximum intensity was 'slight' in five cases, 'moderate' in 15, 'severe' in one and not documented in one case. With regard to these skin reactions, transient treatment modifications, such as reducing the frequency of application or a treatment-free interval of usually a week, were made in 15 patients. In four of these patients the frequency of application was reduced to once daily, in four patients the miltefosine treatment was temporarily stopped and in seven patients both measures were taken. In two patients adverse skin reactions contributed to the decision to discontinue the treatment. Nausea was observed in two patients (6%). It should be noted, however, that the treated area in these two patients was limited (19 $cm^2$ and 2 $cm^2$), which virtually excludes the possibility of a critical systemic uptake of miltefosine. Haematotoxic or blood chemistry changes were not observed.

### Response

Three patients received concomitant systemic tumour therapy and were excluded from response evaluation. Thirty patients were therefore assessable for therapeutic efficacy. The overall response rate (complete response + partial response) was 13/30 (43%), seven of which were complete (23%) and six of which were partial (20%). An example of a patient treated with miltefosine is shown in Figures 2–5. Stable disease was seen in 10/30 patients (33%) and 7/30 (23%) showed progression. The median

© Cancer Research Campaign 1999

**Table 1** Patient characteristics

| Patient characteristics | No. | % |
|---|---|---|
| Total patients | 33 | 100 |
| Evaluable for toxicity | 33 | 100 |
| Evaluable for anti-tumour activity | 30 | 91 |
| Median age, years (range) | 57 (30–90) | |
| WHO performance status | | |
| 0 | 21 | 64 |
| 1 | 10 | 30 |
| 2 | 1 | 3 |
| Not documented | 1 | 3 |
| Pretreatment combination | | |
| Surgery + endocrine therapy | 2 | 6 |
| Surgery + endocrine therapy + chemotherapy | 1 | 3 |
| Surgery + radiation + chemotherapy | 1 | 3 |
| Surgery + radiation + endocrine therapy | 6 | 18 |
| Radiation + endocrine therapy + chemotherapy | 8 | 24 |
| Surgery + radiation + endocrine therapy + chemotherapy | 15 | 45 |
| Time (years) between first diagnosis and start of miltefosine treatment | | |
| ≤ 4 | 14 | 42 |
| 5–8 | 10 | 30 |
| 9–12 | 2 | 6 |
| > 12 | 7 | 21 |
| Time (months) between first occurrence of skin metastases and start of miltefosine treatment | | |
| ≤ 6 | 5 | 15 |
| 7–12 | 2 | 6 |
| 13–24 | 7 | 21 |
| 25–48 | 11 | 33 |
| > 48 | 8 | 24 |

**Figure 2**

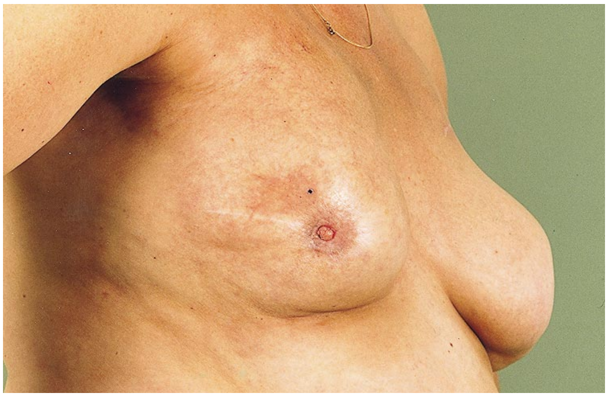

**Figure 3**

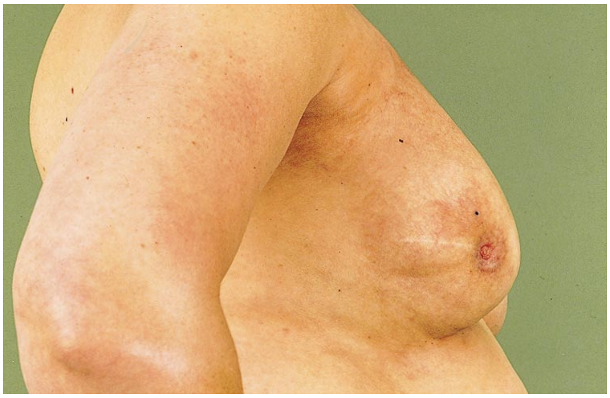

**Figure 4**

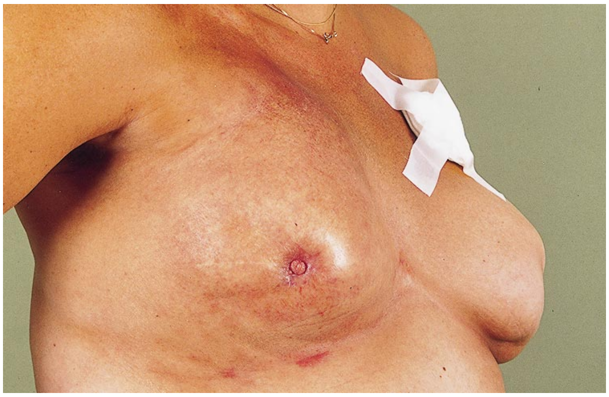

**Figure 5**

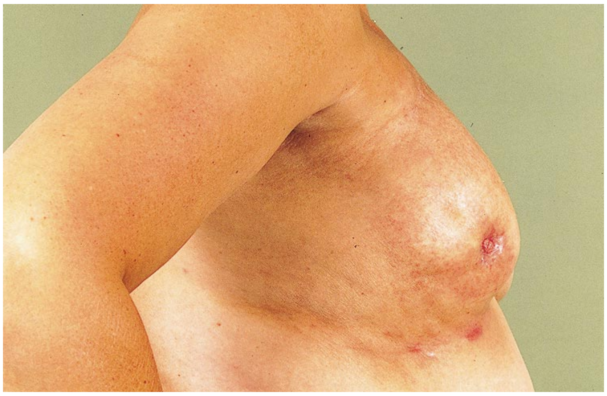

**Figures 2–5** The figures depict skin deposits in a previously irradiated breast of a 53-year-old woman, who presented with inflammatory breast cancer and was treated with anthracycline-based primary chemotherapy. Diagnosis was confirmed by biopsy (black scar in Figures 2 and 3) and she was treated with 8 weeks miltefosine. The treated area shows white discolouration in Figures 4 and 5. Outside the treated area (below the nipple) new skin deposits have developed. These lesions were treated successfully with miltefosine, but other systemic treatment became necessary by rapidly progressive lung metastases. She died 8 months later

*© Cancer Research Campaign 1999*

**Table 2** Response

| Response | Number of patients (%) | Median duration of response in weeks (range) |
|---|---|---|
| Complete remission | 7 (23) | 24 (8–68) |
| Partial remission | 6 (20) | 12 (8–22) |
| Stable disease | 10 (33) | 8 (6–13) |
| Progressive disease | 7 (23) | |

response duration was 18 weeks, range 8–68. In Table 2 response duration is specified according to response.

### DISCUSSION AND CONCLUSION

The topical administration of a cytotoxic drug offers a new approach to the treatment of metastatic skin deposits from breast cancer. A major advantage of this administration route is the reduced risk of systemic toxicity. Furthermore, the drug is easily applied by the patient, eliminating the need for hospitalization (Unger et al, 1990). Several factors such as chemical structure and lipid solubility determine whether a cytotoxic drug will penetrate the skin. Compounds that have a well-balanced water and lipid solubility are in general good penetrants (Unger et al, 1992). Miltefosine is a cytotoxic agent with such favourable characteristics and with proven activity in breast cancer (Hilgard et al, 1988; Eibl and Unger, 1990; Fichtner et al, 1994). To further increase penetration, miltefosine is dissolved in a mixture of short-chain glycerolethers. The resulting oily solution can easily be applied to the skin.

In this study of 33 patients with skin lesions from breast cancer, miltefosine showed promising therapeutic activity. A total response rate (complete response + partial response) of 43% was observed. Comparable results were obtained by Clive and Leonard (1997). The topical administered lotion of miltefosine was frequently (22/33; 67%) accompanied by localized adverse skin reactions but these were usually of mild to moderate intensity (20/22; 91%). Moreover, because these reactions were visible, they were readily managed by reducing the frequency of application (from twice to once daily administration) or by stopping treatment temporarily. Compatible with the results from other studies (Unger et al, 1988, 1990, 1992; Detmar et al, 1994; Clive and Leonard, 1997) we found that systemic toxicity of miltefosine, even after extensive topical treatment, was negligible. Therefore, we conclude that topical administration of miltefosine is an effective treatment to control temporarily breast cancer skin lesions, either in systemically pretreated patients or as a measure to postpone the more aggressive systemic chemotherapy. Under the investigational setting the treatment was discontinued as soon as new (systemic) therapy due to progression of the disease was started. However, continuation of the topical treatment or retreatment in case of relapse might be considered outside clinical trials. The figures in this study are too small to draw a firm conclusion on which lesions are the best suitable for miltefosine treatment; however, flat non-ulcerative intracutaneous lesions seem to respond the best.

### REFERENCES

Ando K, Kodama K, Kato A, Tamura G and Arima K (1972) Antitumor activity of glycerylethers. *Cancer Res* **32**: 125–129

Berkovic D, Grunwald U, Menzel W, Unger C, Hiddeman W and Fleer EA (1995) Effects of hexadecylphosphocholine on membrane phospholipid metabolism in human tumour cells. *Eur J Cancer* **31A**: 2080–2085

Clive S and Leonard RCF (1997) Miltefosine in recurrent cutaneous breast cancer. *Lancet* **349**: 621–622

Detmar M, Geilen CC, Wieder T, Orfanos CE and Reuter W (1994) Phospholipid analogue hexadecylphosphocholine inhibits proliferation and phosphatidylcholine biosynthesis of human epidermal keratinocytes in vitro. *J Inv Derm* **102**: 490–494

Dummer R, Krasovec M, Roeger J, Sindermann H and Burg G (1993) Topical administration of hexadecylphosphocholine in patients with cutaneous lymphomas: results of a phase I/II study. *J Am Academy Derm* **29**: 963–970

Eibl H and Unger C (1990) Hexadecylphosphocholine, a new and selective antitumor drug. *Cancer Treatment Rev* **17**: 233–242

Eibl H, Unger C, Fleer EAM, Kim DJ, Berger MR and Nagel GA (1986) Hexadecylphosphocholine, a new antineoplastic agent: cytotoxic properties in leukaemic cells. *J Cancer Res Clin Oncol* **111**: S24

Fichtner I, Zeisig R, Naundorf H, Jungmann S, Arndt D, Asongwe G, Double JA and Bibby MC (1994) Antineoplastic activity of alkylphosphocholines (APC) in human breast carcinomas in vivo and in vitro; use of liposomes. *Breast Cancer Res Treatment* **32**: 269–279

Goppelt-Struebe M and Winter I (1995) Effects of hexadecylphosphocholine on fatty acid metabolism: relation to cytotoxicity. *Cancer Chemother Pharmacol* **35**: 519–526

Hilgard P, Stekar J, Voegeli R, Engel J, Schumacher W, Eibl H, Unger C and Berger MR (1988) Characterization of the anti-tumor activity of hexadecylphosphocholine. *Eur J Cancer Clin Oncol* **24**: 1457–1461

Hilgard P, Harleman JH, Voegeli R, Maurer HR, Echarti C and Unger C (1989) The antineoplastic activity of hexadecylphosphocholines. *Proc Am Ass Cancer Res* **30**: 2310

Hilgard P, Klenner T, Stekar J and Unger C (1993) Alkylphosphocholines: a new class of membrane-active anticancer agents. *Cancer Chemother Pharm* **32**: 90–95

Planting AST, Stoter G and Verweij J (1993) Phase II study of daily oral miltefosine (hexadecylphosphocholine) in advanced colorectal cancer. *Eur J Cancer* **29A**: 518–519

Unger C and Eibl H (1991) Hexadecylphosphocholine: preclinical and first clinical results of a new antitumor drug. *Lipids* **26**: 1412–1417

Unger C, Eibl H, von Heyden HW and Nagel GA (1984) Reversible opening of the blood-brain-barrier for drugs transfer by short-chain alkyl glycerols. *Proc Am Soc Clin Oncol* **20**: 25

Unger C, von Heyden HW, Breiser A, Nagel CA and Eibl H (1988) Etherlipids in the topical treatment of skin metastases. *J Cancer Res Clin Oncol* **114**: S40

Unger C, Damenz W, Fleer EAM, Kim DJ, Breiser A, Hilgard P, Engel J, Nagel G and Eibl H (1989) Hexadecylphosphocholine, a new ether lipid analogue. *Acta Oncologica* **28**: 213–217

Unger C, Peukert M, Sindermann H, Hilgard P, Nagel G and Eibl H (1990) Hexadecylphosphocholine in the topical treatment of skin metastases in breast cancer patients. *Cancer Treatment Rev* **17**: 243–246

Unger C, Sindermann H, Peukert M, Hilgard P, Engel J and Eibl H (1992) Hexadecylphosphocholine in the topical treatment of skin metastases in breast cancer patients. *Progr Exp Tumor Res* **34**: 153–159

Verweij J, Planting A, van der Burg M and Stoter G (1992) A dose-finding study of miltefosine (hexadecylphosphocholine) in patients with metastatic solid tumours. *J Cancer Res Clin Oncol* **118**: 606–608

Verweij J, Krzemieniecki K, Kok T, Poveda A, van Pottelsberghe C, van Glabbeke M and Mouridsen H (1993) Phase II study of miltefosine in advanced soft tissue sarcomas of the adult – an EORTC soft tissue and bone sarcoma group study. *Eur J Cancer* **29A**: 208–209

Verweij J, Gandia D, Planting AST, Stoter G and Armand JP (1993) Phase II study of oral miltefosine in patients with squamous cell head and neck cancer. *Eur J Cancer* **29A**: 778–779

WHO (1997) *WHO Handbook for Reporting Results of Cancer Treatment*. World Health Organization: Geneva

*© Cancer Research Campaign 1999*
